# Lower vitamin D levels are associated with the pathogenesis of inflammatory bowel diseases

**DOI:** 10.1097/MD.0000000000035505

**Published:** 2023-10-13

**Authors:** Antonia Topalova-Dimitrova, Ivan Valentinov Dimitrov, Rosen Nikolov

**Affiliations:** a Department of Gastroenterology, University Hospital “St. Ivan Rilski”, Sofia, Bulgaria; b Medical University, Sofia, Bulgaria; c Department of Surgery, University Hospital “Tsaritsa Joanna – ISUL”, Sofia, Bulgaria.

**Keywords:** IBD, inflammation, vitamin D

## Abstract

Vitamin D plays a role in regulating immune homeostasis, inflammation and has an impact on the pathogenesis of inflammatory bowel diseases (IBD). IBD has a multifactorial pathogenesis primarily associated with immune dysregulation, dysbiosis, structurally altered intestinal mucosa, and genetic factors. The immunomodulatory function of this vitamin is linked to its control over innate and adaptive immunity, facilitated through its nuclear vitamin D receptor, leading to the inhibition of nuclear factor kappa-B. This study aimed to investigate serum vitamin D levels in patients with IBD compared to healthy individuals and to evaluate the relationship between vitamin D and inflammatory markers. Cross-sectional study. The study included 106 participants divided into 2 groups: patients with IBD (92), and healthy controls (14). The diagnosis of IBD was based on clinical, laboratory, fecal, endoscopic, and histological findings, following the European guidelines for diagnosis and follow-up ECCO-ESGAR guidelines for diagnostic assessment of IBD from 2019. Serum vitamin D levels were measured along with laboratory tests, imaging, and endoscopic examinations. IBD activity was evaluated using the Montreal classification and clinical and endoscopic indices. Data analysis involved calculating the mean, minimum, and maximum values, standard deviation, and Pearson coefficient. The level of statistical significance for this study was set at *P* < .05. The study found a prevalence of vitamin D deficiency in 32.6% of patients with IBD, while 66.3% had insufficiency, as compared with healthy individuals. The mean levels of vitamin D in UC and CD were 16 ± 8.6 ng/mL, whereas in the control healthy group, they were 26 ± 9.73 ng/mL. A statistically significant reverse correlation was observed between lower vitamin D levels and higher levels of the inflammatory markers. The study concluded that IBD patients exhibit lower levels of vitamin D, which is associated with inflammation and may contribute to the pathogenesis of the disease.

## 1. Introduction

The pathogenesis of inflammatory bowel disease is complex and involves a dysfunctional immune reaction to intestinal microflora. Normally, a symbiotic relationship exists between commensal bacteria and the host organism, where resident bacteria reduce the expression of proinflammatory transcription factors, thereby blocking NF-κB activation. It maintains physiological intestinal immune tolerance by suppressing inflammation in response to microorganisms and food antigens. In patients with inflammatory bowel diseases (IBD), immune tolerance is impaired or lost, leading to an inflammatory response triggered by the intestinal microbiota and intraepithelial innate lymphoid cells type 3 (ILC3). This results in structural changes in the mucosal layer of the intestine and increases pathological permeability.^[1]^

The nuclear transcription factor nuclear factor kappa-Bplays a crucial role in maintaining chronic inflammation,^[2]^ and its enhanced activation is strongly correlated with the severity of intestinal mucosal inflammation. Nuclear factor kappa-B activation leads to the synthesis of inflammatory molecules such as tumor necrosis factor-alpha (TNF-α) and various interleukins (IL) such as IL-1, IL-2, IL-6, IL-12, IL-16, and IL-23,^[3]^ which are involved in the pathogenesis of Crohn disease and ulcerative colitis (UC). These interleukins activate antigen-presenting cells and T-lymphocytes Th-1 and Th-17, which maintain chronic inflammation in Crohn disease. In contrast, Th-2 lymphocytes are also involved in UC.^[4]^ Chronic inflammation leads to structural changes in the intestinal mucosa, disrupts the gut barrier function, and may contribute to bacterial translocation and trigger a systemic inflammatory response. The increased gut permeability observed in IBD is associated with damage to epithelial junctions and the stimulated synthesis of zonulin, which leads to changes in the cytoskeleton of cells.^[5]^

Vitamin D plays diverse roles, not only in regulating calcium-phosphate metabolism but also in regulating the immune response, intestinal immunity, and mucosal barrier integrity.^[6]^ The biologically active vitamin D3 binds to its nuclear vitamin D receptor (VDR) found in various tissues, including bone, skeletal muscle, kidney, skin, parathyroid glands, and intestine.^[7]^ VDR is also expressed in immune cells such as monocytes, macrophages, dendritic cells, T-lymphocytes, and B-lymphocytes.^[8]^ Through antigen-presenting cells, vitamin D inhibits the synthesis of inflammatory mediators such as IL-1, IL-6, IL-8, IL-12, and TNF-α, all of which are involved in IBD pathogenesis.^[9]^ It also hinders dendritic cell maturation and T- and B-lymphocyte proliferation, differentiation, and antibody production. By suppressing the maturation of T-lymphocytes, vitamin D and its receptors reduce the population of Th-17 lymphocytes involved in the pathogenesis of Crohn. It also promotes the function of regulatory T cells, which participate in immunosuppression.^[10,11]^

VDR is also expressed on the cell membranes of ILC3, which plays a key role in the development of Crohn disease and UC. The activation of this receptor suppresses ILC3 activity and production of pro-inflammatory cytokines.^[12]^ The integrity of the gut epithelial barrier is maintained by tight junctions, which contain structural proteins, such as claudins and occludins. Vitamin D influences the expression of these proteins, thereby regulating intestinal permeability.^[13]^ Under the influence of proinflammatory molecules such as TNF-α and IL-13, the expression of claudin-2 increases, leading to stimulated paracellular transport and higher gut permeability.^[14]^ Increased claudin-2 synthesis is positively correlated with disease activity.^[13–15]^

A higher prevalence of low serum vitamin D levels (deficiency or insufficiency) has been found among patients with IBD, partly due to malabsorption, bile acid malabsorption (choleretic diarrhea), reduced appetite, limited sunlight exposure due to treatment with azathioprine, corticosteroid therapy, and genetic polymorphisms of VDR.^[16,17]^ Clinical observations have shown that approximately 60% to 64% of patients with Chron disease (CD) and UC exhibit vitamin D deficiency.^[18–21]^ Vitamin D deficiency is associated with a higher rate of disease relapse, increased risk of surgery, and slower response to biological medications.^[22,23]^ Genetic polymorphisms of VDR have been identified in patients with IBD and are associated with lower serum levels and an elevated risk of complications.

## 2. Materials and methods

An observational study was conducted to evaluate serum vitamin D levels in patients with inflammatory bowel disease compared to those in healthy individuals and their statistical correlation with laboratory inflammatory markers. This study included a total of 106 participants. They were divided into 92 patients with Crohn disease and UC and 14 healthy controls. The diagnosis of IBD was based on clinical, laboratory, fecal, endoscopic, and histological findings following the European consensus guidelines for diagnosis and follow-up (ECCO-ESGAR Guideline for Diagnostic Assessment in IBD 2019).^[24]^ All the participants provided informed consent. Demographic data and comorbidities of the patients were evaluated (Table 1).

**Table 1:** Demographic characteristics of patients and comorbidities.

Parameter		IBD	Healthy individuals
Number of participants		n = 92	n = 14
Crohn disease		48	–
Ulcerative colitis		44	–
Gender	Male	46	10
	Female	46	4
Age (x ± SD)		44.2 ± 13.9	42.1 ± 16
	min-max	21–75	19–79
Comorbidities			
1. Arterial hypertension		18	2
2. Diabetes		5	2
3. Coronary artery disease		5	1
4. Metabolic syndrome		10	0
5. Liver cirrhosis		1	0
6. Hepatitis B		1	0
Autoimmune disease		18	1
1. Hashimoto thyroiditis		3	1
2. Ankylosing spondylitis		2	0
3. Rheumatoid arthritis		2	0
4. Autoimmune hepatitis		1	0
5. Primary biliary cholangitis		2	0
6. Reynold syndrome		1	0
7. Systemic lupus		1	0
8. Systemic sclerosis		1	0
9. Psoriatic arthritis		2	0
10. Antiphospholipid syndrome		1	0
11. Vitiligo		1	0
12. Sjogren syndrome		1	0

Values are presented as n (%), mean ± SD, or min–max.

IBD = inflammatory bowel disease, SD = standard deviation.

Laboratory tests included a full blood count, C-reactive protein (CRP)-C, erythrocyte sedimental rate (ESR), fecal calprotectin, blood sugar, insulin, and homeostatic model assessment for insulin resistance. Imaging studies such as abdominal ultrasound and ileocolonoscopy were also conducted. Serum vitamin D levels were measured once a morning on an empty stomach. At the time of testing, none of the participants received vitamin D replacement therapy. Vitamin D levels were classified as follows: deficiency <12 ng/mL, insufficiency between 12 and 30 ng/mL and normal serum level >30 ng/mL. The severity of Crohn disease was assessed using the Crohn disease activity index, and for UC, the Truelove and Witt severity index was used. The extent of IBD was determined using the 2005 Montreal classification.

Data analysis involved calculating the statistical mean value, standard deviation, and Pearson coefficient to evaluate the statistical significance. The “*P* value” was considered statistically significant when *P* < .05.

## 3. Results

**Figure 1.**
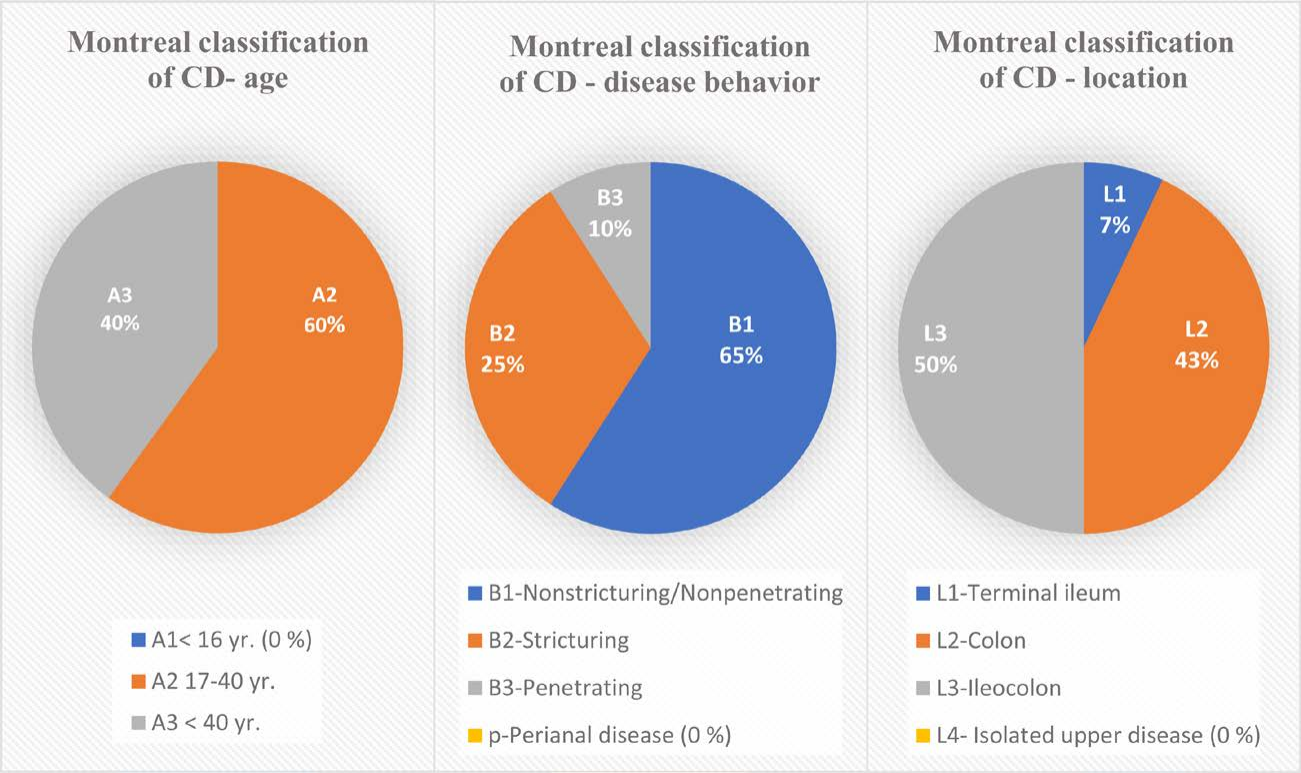
Percentage distribution of included patients with Crohn disease according to the Montreal classification. Based on age they were 60% between 17 and 40 years old (A2) and 40% were above 40 years old (A3). The disease behavior was 65% nonstructuring/penetrating (B1), 25% stricturing (B2), and 10% penetrating (B3). The location of the disease was 50% ileocolonic (L3), 43% with colon involvement (L2) and 7% in the terminal ileum (L1). Values are presented as n (%); CD = Crohn disease; yr = years; age: A1 < 16; A2 17 to 40 yr; A3 < 40 yr; behavior: B1 = nonstricturing/nonpenetrating; B2 = stricturing; B3 = penetrating; p = perianal disease; location: L1 = terminal ileum; L2 = Colon; L3 = ileocolon; L4 = isolated upper disease.

In the conducted study, a total of 48 patients with Crohn were included. According to the Montreal classification, approximately 60% of the patients were between 17 and 40 years of age (A2). The extent of the disease was 50% ileocolonic (L3), and the involvement of the colon was 43% (L2), mainly non-structuring/penetrating (B1) disease behavior (Fig. 1). In this study, none of the patients had CD with upper gastrointestinal involvement or perianal disease. Based on the CD activity index, the largest percentage of patients was in the moderate-to-severe disease activity group (44%), and the mean number of collected points was 249p, followed by severe (33%), mild (14%), and remission (9%).

**Figure 2.**
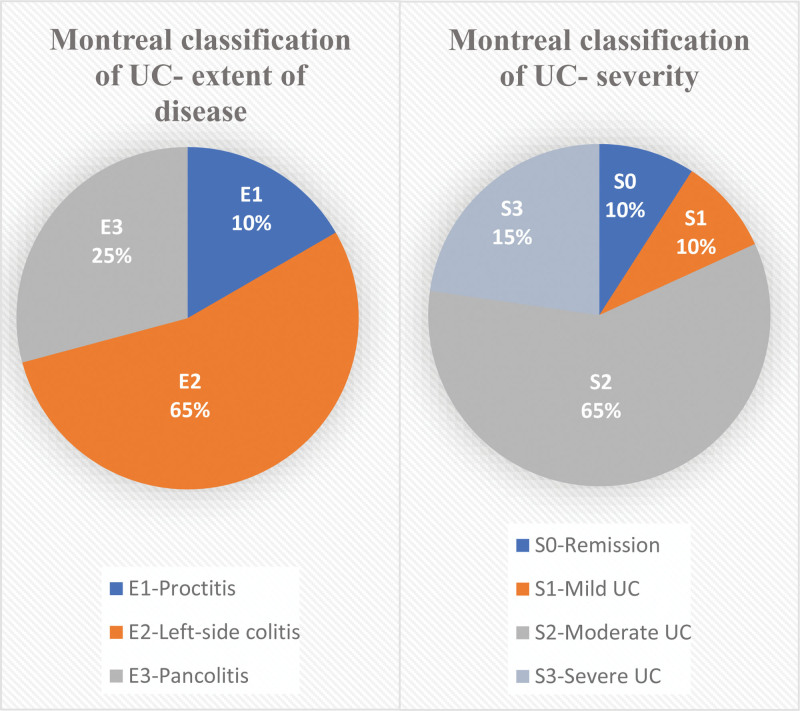
Percentage distribution of individuals with ulcerative colitis according to the Montreal classification who participated in the study. The extent of the disease was 65% left-sided colitis (E2), 25% pancolitis (E3), and 10% proctitis (E1). Based on the severity of the disease 65% are with moderate (S2), 15% severe (S3), 10 % were in remission (S0), and 10% had mild (S1) severity. Values are presented as n (%); UC = ulcerative colitis; extent: E1 = proctitis; E2 = left-side colitis; E3 = pancolitis; severity: S0 = remission; S1 = mild UC; S2 = moderate UC; S3 = severe UC.

This cross-sectional study included 44 individuals with UC. Disease location and severity were evaluated using the 2005 Montreal classification. Most cases were left-sided colitis (65%, E2), pancolitis (25%, E3) and the severity of the disease was mostly moderate at (65%, S2) shown in (Fig. 2). Based on Truelove and Witt severity index of UC, we found a higher percentage of patients with mild and moderate severity of the disease (45%), severe activity (30%), and mild (25%).

All IBD patients in the study had taken at least once corticosteroids at the time of establishing the diagnosis. Individuals with UC were divided into 2 groups according to their medication: patients on biological therapy n = 20 and those without (n = 22). Most patients were taking only 5-aminosalycilic acid (33%), followed by adalimumab (23%), azathioprine (22%), infliximab and vedolizumab (10%), and certolizumab pegol (2%). Patients with CD receiving biological treatment were n = 28 and those without were n = 20. The main therapies in these groups were adalimumab alone (25%), azathioprine (22%), 5-aminosalycilic acid (18%), vedolizumab (14%), ustekinumab (12%), and infliximab (9%). Among all IBD patients, the urgency to take corticosteroids during the study and by the time of examining vitamin D levels were 2 individuals with CD and one with UC due to relapse.

The laboratory results of all individuals are presented in Table 2. Inflammatory markers, which are associated with disease activity, are significantly higher in IBD patients than in healthy controls. Transferrin saturation was expectedly lower in IBD patients (23 ± 11%) than in healthy participants (35 ± 8%), possibly because of factors such as malabsorption, reduced appetite, increased hepcidin synthesis during chronic inflammation, and bleeding. The serum vitamin D levels in patients with CD and UC were 16 ± 8.6 ng/mL, classifying them as deficient or insufficient in vitamin D. Healthy individuals had significantly higher values at 26 ± 9.73 ng/mL (Table 2). Among IBD patients, 32.6% had vitamin D deficiency, 66.3% had insufficiency, and only 1.08% had normal serum levels. In healthy controls, 35.7% had normal serum levels, 57.1% had insufficiency, and 7% had deficiency.

**Table 2:** Laboratory markers in patients with IBD and healthy individuals.

Parameter	Referral range	IBD	Healthy individuals
WBC	3.5–10.80 × 10^9^/L	8.71 ± 3.15 3.6–22.8	7.2 ± 2.5 4.08–13.8
Hb	120–160 g/L	135.6 ± 20.1 175–70	145 ± 15 120–176
Pt	130–36 × 10^9^/L	331 ± 123.7 165–840	249 ± 61 145–367
Transferrin saturation	>20%	23 ± 11 2–58	35 ± 8 25–54
ESR	0–25 mm/h	19.3 ± 19 1–80	9 ± 10 2–60
CRP	0–6 mg/L	18 ± 59 1–445	4 ± 2.7 0.6–10
Fecal calprotectin	Cut off −250 mg/kg	473 ± 317 21–1000	64 ± 42.16 20–150
Homa-IR	<2.5 insulin resistance	2.6 ± 2.6 0.1–12.7	1.96 ± 1.76 0.5–7.5
Vitamin D	Deficiency <12 ng/mL Insufficiency 12–30 nL/mL Normal value >30 ng/mL	16 ± 8.6 5–33	26 ± 9.73 14–53

Values are presented as n (%), mean ± SD, or min–max.

CRP = C-reactive protein, ESR = erythrocyte sedimentation rate, Hb = hemoglobin, Homa-IR = homa index, IBD = inflammatory bowel disease, Pt = platelets, WBC = white blood cell.

Statistical analysis showed a reverse correlation between serum vitamin D levels and leukocyte counts in patients with IBD (correlation coefficient of −0.282, *P* = .006). A similar negative correlation was observed between vitamin D and CRP-C values (coefficient −0.288, *P* value .005).

A negative correlation between vitamin D levels and ESR was found with a coefficient of −0.260 and *P* value of .012. A reverse correlation was found between fecal calprotectin and vitamin D, with a coefficient of −0.292 and a significance level of 0.005. The strongest correlations were found between vitamin D level, leukocyte count, and CRP-C.

## 4. Discussion

Vitamin D is a steroid hormone with immunomodulatory activity. Low levels of IBD are commonly observed in patients with IBD due to malabsorption, reduced food intake, choleretic diarrhea, limited sunlight exposure while on immunosuppressants and corticosteroids, and single gene polymorphisms of the VDR. A combination of impaired vitamin D signaling, genetic predisposition, and environmental factors may contribute to the development of autoimmunity. Vitamin D deficiency in patients with CD and UC is associated with a higher frequency of disease relapse, slower response to biological therapy, and increased risk of surgical intervention.

In our clinical observations, we found a higher rate of vitamin D deficiency (32.6%) and insufficiency (66.3%) in IBD patients. Most patients with CD exhibit moderate-to-severe disease activity and ileocolonic location. Patients with UC predominantly have mild to moderate severity and left-sided colitis. At the time of examination only 3 patients were taking corticosteroids alongside with biologics or immunosuppressants.

A statistically significant negative relationship was observed between vitamin D levels and inflammatory markers, including the ESR, CRP, fecal calprotectin, leukocytes, and platelets. Lower vitamin D levels are associated with higher levels of inflammatory markers. These findings confirm the association between vitamin D levels and inflammation.

The present study had some limitations. First, due to its cross-sectional nature, the collected data are at a single point in time, making it challenging to establish causality of lower vitamin D levels. Second, these types of studies provide information about prevalence, which is the proportion of cases in a population, but do not provide information about incidence. Third, the number of healthy individuals is limited owing to time and equipment restrictions, as well as the cost of examinations. However, this study revealed the potential role of vitamin D in patients with Chron and UC.

## 5. Conclusion

This study evaluated the tendency of vitamin D deficiency and insufficiency in patients with IBD and revealed a statistically significant reverse correlation with higher inflammatory markers. The assessment of vitamin D status in patients with IBD is firmly recommended because of the potential risk of relapse, surgical treatment, development of osteoporosis, calcium deficiency, and slower response to biologics. An increase in vitamin D levels could be achieved by improvement of diet or increased sun exposure, which is problematic for people with IBD, or by oral supplementation.

## Acknowledgments

We are extremely grateful to all the physicians who contributed to the collection of this data and the participants for participating in the clinical study.

## Author contributions

**Conceptualization:** Antonia Topalova-Dimitrova, Ivan Valentinov Dimitrov, Rosen Nikolov.

**Data curation:** Antonia Topalova-Dimitrova.

**Investigation:** Antonia Topalova-Dimitrova, Ivan Valentinov Dimitrov.

**Supervision:** Rosen Nikolov.

**Writing – original draft:** Antonia Topalova-Dimitrova.

**Writing – review & editing:** Antonia Topalova-Dimitrova.

## References

[1] Shih DQ Targan SR. Immunopathogenesis of inflammatory bowel disease. World J Gastroenterol. 2008;14:390–400. 18200661 10.3748/wjg.14.390 PMC2679127

[2] Mitchell S Vargas J Hoffmann A. Signaling via the NFκB system. Wiley Interdiscip Rev Syst Biol Med. 2016;8:227–41. 26990581 10.1002/wsbm.1331 PMC8363188

[3] Becker C Wirtz S Ma X. Regulation of IL-12 p40 promoter activity in primary human monocytes: roles of NF-kappaB, CCAAT/enhancer-binding protein beta, and PU.1 and identification of a novel repressor element (GA-12) that responds to IL-4 and prostaglandin E(2). J Immunol. 2001;167:2608–18. 11509602 10.4049/jimmunol.167.5.2608

[4] Strober W Fuss IJ. Proinflammatory cytokines in the pathogenesis of inflammatory bowel diseases. Gastroenterology. 2011;140:1756–67. 21530742 10.1053/j.gastro.2011.02.016 PMC3773507

[5] El Asmar R Panigrahi P Bamford P. Host-dependent zonulin secretion causes the impairment of the small intestine barrier function after bacterial exposure [published correction appears in Gastroenterology 2003 Jan;124(1):275. El Asmar Rahzi [corrected to El Asmar Ramzi]]. Gastroenterology. 2002;123:1607–15. 12404235 10.1053/gast.2002.36578

[6] Chirumbolo S Bjørklund G Sboarina A. The Role of Vitamin D in the immune system as a pro-survival molecule. Clin Ther. 2017;39:894–916. 28438353 10.1016/j.clinthera.2017.03.021

[7] Dowd DR MacDonald P. Vitamin D Receptor, Encyclopedia of Biological Chemistry (Second Edition), 2013

[8] Aranow C. Vitamin D and the immune system. J Investig Med. 2011;59:881–6. 10.231/JIM.0b013e31821b8755 PMC3166406 21527855

[9] Almerighi C Sinistro A Cavazza A. 1Alpha,25-dihydroxyvitamin D3 inhibits CD40L-induced pro-inflammatory and immunomodulatory activity in human monocytes. Cytokine. 2009;45:190–7. 19186073 10.1016/j.cyto.2008.12.009

[10] Grover P Goel PN Greene MI. Regulatory T cells: regulation of identity and function. Front Immunol. 2021;12:750542. 34675933 10.3389/fimmu.2021.750542 PMC8524049

[11] Konya V Czarnewski P Forkel M. Vitamin D downregulates the IL-23 receptor pathway in human mucosal group 3 innate lymphoid cells. J Allergy Clin Immunol. 2018;141:279–92. 28433688 10.1016/j.jaci.2017.01.045

[12] Anderson JM. Molecular structure of tight junctions and their role in epithelial transport. News Physiol Sci. 2001;16:126–30. 11443232 10.1152/physiologyonline.2001.16.3.126

[13] Prasad S Mingrino R Kaukinen K. Inflammatory processes have differential effects on claudins 2, 3 and 4 in colonic epithelial cells. Lab Invest. 2005;85:1139–62. 16007110 10.1038/labinvest.3700316

[14] Luettig J Rosenthal R Barmeyer C. Claudin-2 as a mediator of leaky gut barrier during intestinal inflammation. Tissue Barriers. 2015;3:e977176. 25838982 10.4161/21688370.2014.977176 PMC4372021

[15] Weber CR Nalle SC Tretiakova M. Claudin-1 and claudin-2 expression is elevated in inflammatory bowel disease and may contribute to early neoplastic transformation. Lab Invest. 2008;88:1110–20. 18711353 10.1038/labinvest.2008.78 PMC2586671

[16] Zhang YG Wu S Lu R. Tight junction CLDN2 gene is a direct target of the vitamin D receptor. Sci Rep. 2015;5:10642. 26212084 10.1038/srep10642 PMC4650691

[17] Shirwaikar Thomas A Criss ZK Shroyer NF. Vitamin D receptor gene single nucleotide polymorphisms and association with Vitamin D levels and endoscopic disease activity in inflammatory bowel disease patients: a pilot study. Inflamm Bowel Dis. 2021;27:1263–9. 33165606 10.1093/ibd/izaa292 PMC8785942

[18] Del Pinto R Pietropaoli D Chandar AK. Association between inflammatory bowel disease and Vitamin D deficiency: a systematic review and meta-analysis. Inflamm Bowel Dis. 2015;21:2708–17. 26348447 10.1097/MIB.0000000000000546 PMC4615394

[19] Burrelli Scotti G Afferri MT De Carolis A. Factors affecting vitamin D deficiency in active inflammatory bowel diseases. Dig Liver Dis. 2019;51:657–62. 30587439 10.1016/j.dld.2018.11.036

[20] Caviezel D Maissen S Niess JH. High prevalence of Vitamin D deficiency among patients with inflammatory bowel disease. Inflamm Intest Dis. 2018;2:200–10. 30221147 10.1159/000489010 PMC6135223

[21] Rasouli E Sadeghi N Parsi A. Relationship between Vitamin D deficiency and disease activity in patients with inflammatory bowel disease in Ahvaz, Iran. Clin Exp Gastroenterol. 2020;13:419–25. 33061520 10.2147/CEG.S254278 PMC7537799

[22] Dolatshahi S Pishgar E Jamali R. Does serum 25 hydroxy vitamin D level predict disease activity in ulcerative colitis patients? Acta Clin Belg. 2016;71:46–50. 27075790 10.1080/17843286.2015.1110895

[23] Kabbani TA Koutroubakis IE Schoen RE. Association of Vitamin D level with clinical status in inflammatory bowel disease: a 5-Year longitudinal study. Am J Gastroenterol. 2016;111:712–9. 26952579 10.1038/ajg.2016.53

[24] Maaser C Sturm A Vavricka SR. ECCO-ESGAR guideline for diagnostic assessment in IBD Part 1: initial diagnosis, monitoring of known IBD, detection of complications. J Crohns Colitis. 2019;13:144–64. 30137275 10.1093/ecco-jcc/jjy113

